# Differential Abnormality in Regional Brain Spontaneous Activity and Functional Connectivity in Patients of Non‐Acute Subcortical Stroke With Versus Without Global Cognitive Functional Impairment

**DOI:** 10.1002/brb3.70356

**Published:** 2025-02-25

**Authors:** Yao Wang, Wan Liu, Wenjie Yang, Xue Chai, Hao Yu, Hongxia Ma, Li Liu, Jiang Rao, Guangxu Xu, Zhibin Hu

**Affiliations:** ^1^ Department of Rehabilitation Medicine The Affiliated Brain Hospital of Nanjing Medical University Nanjing Jiangsu Province China; ^2^ Department of Radiology The Affiliated Brain Hospital of Nanjing Medical University Nanjing Jiangsu Province China; ^3^ Institution of Brain Functional Imaging Nanjing Medical University Nanjing Jiangsu Province China; ^4^ Department of Biostatistics, School of Public Health Nanjing Medical University Nanjing Jiangsu Province China; ^5^ Department of Epidemiology, Center for Global Health, School of Public Health Nanjing Medical University Nanjing Jiangsu Province China; ^6^ Rehabilitation Medicine Center The First Affiliated Hospital of Nanjing Medical University Nanjing Jiangsu Province China

**Keywords:** cognitive impairment, functional magnetic resonance imaging, stroke

## Abstract

**Introduction:**

Cognitive impairment after a stroke significantly affects patients' quality of life, yet not all strokes lead to such impairment, and the underlying reasons remain unclear. This study employs resting‐state functional magnetic resonance imaging (rs‐fMRI) to compare subcortical stroke patients with and without cognitive impairment. Our goal is to identify distinct abnormalities in regional brain spontaneous activity and functional connectivity (FC) to better understand the neural basis of post‐stroke cognitive outcomes.

**Methods:**

A total of 62 first‐ever non‐acute subcortical stroke patients were classified into post‐stroke with abnormal cognition (PSAC) and with normal cognition (PSNC) groups. Rs‐MRI was utilized to assess regional homogeneity (ReHo) in 32 PSAC, 30 PSNC, and 62 age‐ and sex‐matched healthy controls (HC). Then we performed the seed‐based whole‐brain FC analysis based on the ReHo results. A partial correlation analysis examined the relationship between altered ReHo or FC and Montreal Cognitive Assessment (MoCA) scores.

**Results:**

It showed varied activity in cognitive‐related brain regions in both stroke groups compared to HC, such as the right superior frontal gyrus, the right middle temporal gyrus, the right postcentral gyrus, and the left cerebellar lobules. The PSAC group had increased activity in the bilateral inferior temporal gyrus as well. Significant differences in activity were also found between PSAC and PSNC groups, with the PSAC group showing decreased activity in the left gyrus rectus (REC) and increased activity in cerebellar lobules. FC analysis revealed decreased connections in the PSAC group, particularly involving the left REC. Activity and FC in left REC and cerebellum also significantly correlated with MoCA scores.

**Conclusions:**

These findings suggest unique patterns of brain activity and connectivity in non‐acute subcortical stroke patients with cognitive impairment, shedding light on potential neural mechanisms underlying post‐stroke cognitive impairment. While the left REC may be a potential neural regulatory stimulus target in clinical applications.

## Graphical abstract

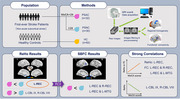

## 1 Introduction

Cognitive impairment or decline is prevalent in over two‐thirds of stroke survivors post‐event (Qu et al. 2015; Aben et al. 2018), leading to challenges in executing daily activities and potentially signaling an unfavorable prognosis such as suboptimal rehabilitation results, heightened mortality rates, and the potential development of dementia (Leys et al. 2005; Nys et al. 2006). Subcortical strokes, particularly those affecting regions such as the thalamus, basal ganglia, and white matter tracts, often disrupt essential neural networks (Fox 2018) and long‐range brain connections, despite the preservation of primary cortical regions (Weaver et al. 2021). Disruptions within these networks and connections can impair cognitive functions ranging from memory and attention to executive function and processing speed (Liu et al. 2017). Nevertheless, the precise changes in spontaneous brain activity and functional connectivity (FC) that differentiate cognitively impaired stroke patients from those who maintain cognitive function have not been thoroughly investigated.

Resting‐state functional magnetic resonance imaging (rs‐fMRI) has gained popularity for exploring cognitive impairment neural mechanisms (Scherr et al. 2021; Dacosta‐Aguayo et al. 2014). Growing investigators employ regional homogeneity (ReHo), which measures the temporal synchronization of the nearest neighbor time series and can map local spontaneous neural activity (Zang et al. 2004), for exploring the local neural activities in cerebrovascular diseases. A previous study (Ni et al. 2016) found decreased ReHo in the precuneus/cuneus and insula; and increased ReHo in the temporal lobe in patients with mild cognitive impairment (MCI) with lacunar infarction (LI), when compared to those without LI. Similarly, post‐stroke patients with cognitive dysfunction revealed decreased ReHo in the anterior cingulate cortices (ACC) and posterior cingulate cortices (PCC), and positively correlated with the cognition scores, when compared to those without cognition decline (Peng et al. 2016). Vascular cognitive impairment (VCI) patients exhibit a significant positive correlation between the ReHo value in the middle temporal gyrus (MTG) and short‐term delayed memory scores (Cai et al. 2023).

ReHo is referred to as short‐term connectivity, while FC could reflect the time consistency of spontaneous activity between regions and could be used to map long‐term connectivity (Peng et al. 2016). Previous studies revealed a significant decrease in the default mode network (DMN) and salience network (Wang, Rao, Chen, et al. 2021), while others reported increased brain activity in DMN, frontal, frontotemporal, and secondary visual network in stroke patients (Dacosta‐Aguayo et al. 2014). They further found significant impairment in the connectivity including superior frontal gyrus (SFG), PCC, parahippocampal gyrus (PHG), and parietal gyrus (Dacosta‐Aguayo et al. 2015).

Nevertheless, the distribution of brain regions and the correlation and value with cognitive impairment were different, possibly due to the selection of subjects and controls. Meanwhile, we found patients who do not acquire cognitive abnormalities after stroke are not adequately studied for brain changes by fMRI when compared to cognitively impaired and healthy people. Besides, there is currently a paucity of knowledge regarding the combination of brain regional activity and further connectivity analysis. Understanding the differential abnormalities in regional brain spontaneous activity and FC between patients with non‐acute subcortical stroke with versus without cognitive impairment could offer valuable insights into the neural underpinnings for post‐stroke cognitive impairment, potentially guiding the development of targeted therapeutic strategies.

Hence, we employ rs‐fMRI to investigate the changes of ReHo in stroke patients with and without cognitive impairment. Then, to study the long‐term FC patterns in these patients based on the regions where the changed ReHo is situated, considered as the regions of interest (ROIs). We hypothesize that (1) distinctive patterns of damage may exist in non‐acute subcortical stroke survivors with and without cognitive impairment and that (2) these different patterns of damage can be captured by rs‐fMRI, specifically in terms of different regional brain spontaneous activity and in the coherence of activity between brain regions, and are closely related to clinical cognitive performance.

## 2 Materials and Methods

### 2.1 Subjects

In this study, 129 subjects were recruited: 67 stroke patients and 62 healthy controls (HC), from Nanjing Brain Hospital between November 2021 and May 2023. Healthy volunteers, recruited via advertisements, were matched to stroke patients by age and gender. Inclusion criteria for stroke patients were: (1) adults (18–80 years old); (2) right‐handedness before stroke; (3) first‐ever ischemic or hemorrhagic stroke confirmed by CT or MRI in the supratentorial subcortical region; (4) at least 2 weeks post‐stroke onset. Exclusion criteria for stroke groups included: (1) a previous history of cognitive impairment before stroke onset; (2) neurodegenerative diseases, epilepsy, mood disorders, traumatic brain injury, or tumors; (3) severe white matter hyperintensity manifesting as a Fazekas scale score > 1 (Fazekas et al. 1987); (4) significant or unstable medical disorders; (5) psychiatric medications affecting cognitive function; and (6) MRI contraindications. Control subjects with silent brain infarctions or cognitive disorders were excluded.

The study was approved by the Ethics Committee of The Affiliated Brain Hospital to Nanjing Medical University (No. 2022‐KY086‐01). All participants provided written informed consent.

### 2.2 Clinical Data, Neuropsychological Tests, and Grouping

Education years, disease duration since stroke onset to the date of fMRI scanning and scale assessing, stroke type, lesion volume, National Institute of Health Stroke Scale (NIHSS) scores, and Fazekas scale scores were obtained through surveys and medical records. Global cognitive function was assessed using the MoCA (Ismail et al. 2010). Stroke survivors were categorized into post‐stroke with abnormal cognition (PSAC) and post‐stroke with normal cognition (PSNC) subgroups based on MoCA scores, with an adjusted score of less than 24 indicating cognitive impairment (Tiffin‐Richards et al. 2014). The Barthel Index (BI) is used to assess stroke patients' activities of daily living.

### 2.3 Brain MRI Data Acquisition

MRI acquisition parameters for all individuals were summarized in Supporting Material.

### 2.4 MRI Data Analysis

#### 2.4.1 Image Preprocessing

Data Processing Assistant for Resting‐State fMRI (DPARSF; http://www.restfmri.net/forum/DPARSF) was employed to preprocess data utilizing Statistical Parametric Mapping (SPM8) (http://www.fil.ion.ucl.ac.uk/spm) in MATLAB environment (Mathworks, Natick, MA, USA). The first 10 volumes of functional images were removed for each patient. Then, the remaining images were corrected using slice‐timing and realignment, accounting for head motion, normalized to standard space using DARTEL, resampled to a 3 × 3 × 3 mm^3^ voxel size, regress nuisance variable, and spatially smoothed with 6 mm full width at half maximum (FWHM). The nuisance variables include 24 motion parameters (six head motion parameters, six head motion parameters one‐time point before, and the 12 corresponding squared items), a global signal, a white matter signal, and a cerebrospinal fluid signal. Finally, a temporal filter (0.01–0.08 Hz) was applied to reduce low‐frequency drift and high‐frequency physiological noise. In addition, patients with excessive head motion (cumulative translation or rotation > 3.0 mm or 3°) were excluded (Chen et al. 2016).

#### 2.4.2 ReHo Analysis

ReHo analysis was carried out on the previously mentioned preprocessed pictures. Calculating Kendall's coefficient of concordance of a voxel's time series with its nearest neighbors (26 voxels) produced individual ReHo maps (Zang et al. 2004). Each voxel's ReHo was normalized to the global mean to reduce individual variances. Finally, all the subjects were smoothed using a 6‐mm FWHM Gaussian kernel.

#### 2.4.3 FC Analysis

The clusters of the between‐group differences in ReHo were set as seeds (region of interest, ROI) for further seed‐based whole‐brain FC analysis (Wang, Rao, Yu et al. 2021). Individual mean time series were extracted based on the coregistered seed region as the reference time series. The correlation analyses were conducted on the seed region and the whole brain within the gray matter mask. We used Fisher's *r*‐to‐*z* transformation to improve the normality of the correlation coefficients. Therefore, an entire brain *z*‐score map was developed for each subject for subsequent statistical analyses.

### 2.5 Statistical Analysis

One‐way analysis of variance (ANOVA) and *χ* ^2^ test were used to compare demographics and cognitive scores between the stroke groups and the HC group using SPSS Statistics 25.0 on continuous variables and proportions, respectively. Statistical significance was determined at *p* < 0.05. A one‐way analysis of covariance (ANCOVA) was used to compare ReHo and the seed‐based FC among the three groups, and a two‐sample *t*‐test was used as a post hoc analysis for the significant clusters of the between‐group differences. Age, gender, education, head motion parameters, and grey matter volume were covariables for the three groups. For stroke groups, we added lesion volume, side, location, disease duration, scores of NIHSS and Fazekas, and stroke type as covariates. The resultant T‐maps were conducted with Gaussian random field theory (GRF) correction for multiple comparisons with voxel *p* < 0.01 and cluster *p* < 0.05 (Quan et al. 2022). Finally, extracted ReHo or FCs of significantly changed regions were used for correlation analysis. After controlling age, gender, and education, partial correlation analyses revealed associations between altered ReHo or FCs and MoCA scores.

## 3 Results

### 3.1 Demographic and Neuropsychological Characteristics

Three PSAC and two PSNC participants were excluded due to excessive head movement. The final enrolment was 62 stroke patients (32 PSAC, 30 PSNC). Table 1 shows participant characteristics. Age and gender were comparable among the three groups. Duration since stroke onset to date of fMRI scanning and scale assessing, NIHSS, Fazekas and BI scores, the lesion side, lesion volume, and distribution were comparable between the two stroke groups. Overlaps of lesions were provided in Figure S1. The PSAC group had fewer years of education (9.56 ± 4.27) than the HC (12.50 ± 2.76, *p* < 0.001) and PSNC (12.53 ± 3.38, *p* < 0.001) groups. Stroke types differed significantly between PSAC (Ischemic/Hemorrhagic: 18/14) and PSNC (I/H: 26/4, *p* = 0.008). Most ischemic cases were large‐artery atherosclerosis (Table S1), and all hemorrhagic cases were spontaneous. MoCA scores were significantly lower in PSAC (11.31 ± 5.28) compared to HC (25.76 ± 1.90, *p* < 0.001) and PSNC (26.60 ± 1.81, *p* < 0.001).

**TABLE 1:** Demographic characteristics of stroke patients and healthy controls.

	HC (*n* = 62)	All SPs (*n* = 62)	PSAC (*n* = 32)	PSNC (*n* = 30)	*F/t/χ* ^2^	*p* value
Gender (M/F), *n*	36/26	37/25	18/14	19/11	0.033^a^, 0.354^b^	0.855^a^, 0.838^b^
Age (mean ± SD), years	61.95 ± 6.56	60.61 ± 10.21	62.59 ± 10.09	58.50 ± 10.08	0.869^a^, 2.184^b^	0.387^a^, 0.117^b^, 0.729^c^, 0.070^d^, 0.060^e^
Education (mean ± SD), years	12.50 ± 2.76	11.00 ± 4.12	9.56 ± 4.27	12.53 ± 3.38	2.382^a^, 9.163^b^	0.019^a*^, <0.001^b*^, <0.001^c*^, 0.964^d^ , <0.001^e*^
Duration_fMRI (mean ± SD), days		48.35 ± 49.71	46.94 ± 51.25	49.87 ± 48.84	0.230	0.819
Duration_Scale (mean ± SD), days	—	45.63 ± 49.13	44.16 ± 50.67	47.20 ± 48.26	0.242	0.809
Stroke type (ischemic/ hemorrhagic)	—	44/18	18/14	26/4	6.953^e^	0.008^e*^
Lesion volume (mean ± SD), mL	—	6.75 ± 10.83	9.21 ± 14.12	4.12 ± 4.46	−1.888^e^	0.064^e^
Lesion side (left/right), *n*		29/33	17/15	12/18	1.071	0.301
Lesion location, *n*					4.737	0.192
Corona radiate		7	4	3		
Basal ganglia		42	23	19		
Paraventricular region		11	3	8		
Thalamus		2	2	0		
NIHSS score (mean ± SD)	—	6.40 ± 2.68	6.34 ± 2.61	6.47 ± 2.79	0.179	0.858
Fazekas score (0/1), *n*	—	11/51	7/25	4/26	0.774	0.379
MoCA score (mean ± SD)	25.76 ± 1.90	18.71 ± 8.66	11.31 ± 5.28	26.60 ± 1.81	6.258^a^, 264.621^b^	**<0.001** ^a^ ^*^, < 0.001^b*^, < 0.001^c*^, 0.228^d^, < 0.001^e*^
BI (mean ± SD)		61.53 ± 19.30	59.06 ± 20.18	64.17 ± 18.29	1.041	0.302

Abbreviations: BI: Barthel Index; Duration_fMRI: disease duration from stroke onset to fMRI scanning date; Duration_Scale: disease duration from stroke onset to scaling date; F: female; HC: healthy controls; M: male; MoCA: Montreal Cognitive Assessment; NIHSS: National Institutes of Health Stroke Scale; PSAC: post‐stroke with

abnormal cognition; PSNC: post‐stroke with normal cognition; SP: stroke patients.

^a^Represents comparison of HC versus all stroke patients.

^b^Represents comparison in HC, PSAC, and PSNC groups.

^c^Represents comparison of HC versus PSAC.

^d^Represents comparison of HC versus PSNC.

^e^Represents comparison of PSAC versus PSNC.

^*^Significant differences were found, *p* < 0.05.

### 3.2 Comparisons of ReHo Values Among the Groups of PSAC, PSNC, and HC

**FIGURE 1:**
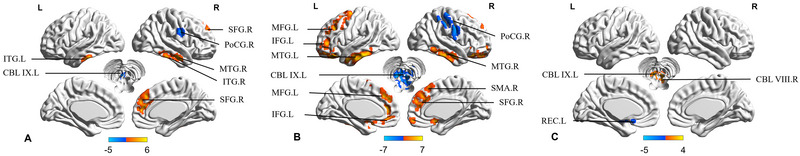
Differences in ReHo among the three groups. Regions exhibiting substantial differences in ReHo values of (A) PSAC versus HC group, (B) PSNC versus HC group, and (C) PSAC versus PSNC group. The resultant T‐maps were conducted with Gaussian random field theory (GRF) correction for multiple comparisons with voxel *p* < 0.01, cluster *p* < 0.05, and cluster size > 30 voxels. CBL: cerebellar lobules; HC: healthy controls; IFG: inferior frontal gyrus; ITG: inferior temporal gyrus; MFG: middle frontal gyrus; MTG: middle temporal gyrus; PoCG: postcentral gyrus; PSAC: post‐stroke with abnormal cognition; PSNC: post‐stroke with normal cognition; REC: gyrus rectus; ReHo: regional homogeneity; SFG: superior frontal gyrus; SMA: supplementary motor area.

ANCOVA revealed significant ReHo differences among the three groups (Figure S2; Table S2). Both stroke groups had increased ReHo in the right SFG and right MTG and decreased ReHo in the right postcentral gyrus (PoCG) and left cerebellar lobules (CBL) IX, compared to the HC group. PSAC showed higher ReHo in the bilateral inferior temporal gyrus (ITG) (Figure 1A; Table 2). PSNC had increased ReHo in the left MTG, left middle frontal gyrus (MFG), left inferior frontal gyrus (IFG), and right supplementary motor area (SMA) (Figure 1B; Table 2). PSAC also had reduced ReHo in the left gyrus rectus (REC) but increased ReHo in left CBL IX and right CBL VIII compared to PSNC (Figure 1C; Table 2).

**TABLE 2:** The differences in the brain regions of ReHo by post hoc analyses among the three groups.

Brain region (AAL)	Peak MNI coordinate			Peak T‐value	Cluster size
	*x*	*y*	*z*		
**PSAC < HC**					
Left cerebellar lobules IX	−3	−48	−48	−4.2431	117
Right postcentral gyrus	45	−6	27	−4.0183	50
**PSAC > HC**					
Right middle temporal gyrus	60	−12	−18	4.5295	88
Left inferior temporal gyrus	−63	−30	−24	4.0988	90
Right inferior temporal gyrus	66	−36	−18	5.2676	82
Right superior frontal gyrus	9	39	36	4.8463	103
**PSNC < HC**					
Left cerebellar lobules IX	−12	−45	−54	−6.0934	811
Right postcentral gyrus	66	−12	33	−5.0586	243
**PSNC > HC**					
Left middle temporal gyrus	−60	−15	−24	6.1397	340
Right middle temporal gyrus	60	−12	−18	5.3146	314
Left inferior frontal gyrus	−18	18	−18	6.0293	529
Right superior frontal gyrus	18	24	18	5.8441	294
Left middle frontal gyrus	−33	21	42	6.0466	423
Right supplementary motor area	9	21	54	4.4843	125
**PSAC < PSNC**					
Left gyrus rectus	−9	15	−15	−4.2535	76
**PSAC > PSNC**					
Left cerebellar lobules IX	−9	−54	−63	3.9683	72
Right cerebellar lobules VIII	9	−42	−60	3.9663	148

*Note*: The resultant T‐maps were conducted with Gaussian random field theory (GRF) correction for multiple comparisons with voxel *p* < 0.01, cluster *p* < 0.05, and cluster size > 30 voxels.

Abbreviations: AAL: automated anatomical labeling; HC: healthy controls; MNI: Montreal Neurological Institute; PSAC: post‐stroke with abnormal cognition; PSNC: post‐stroke with normal cognition; ReHo: regional homogeneity.

### 3.3 Comparison of FC Among the Groups of PSAC, PSNC, and HC

**FIGURE 2:**
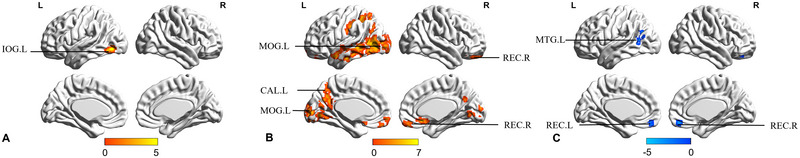
Differences in FC based on ROIs of left REC among the three groups. Regions exhibiting substantial differences in FC values of (A) PSAC versus HC group, (B) PSNC versus HC group, and (C) PSAC versus PSNC group. The resultant T‐maps were conducted with Gaussian random field theory (GRF) correction for multiple comparisons with voxel *p* < 0.01, cluster *p* < 0.05, and cluster size > 30 voxels. CAL: Calcarine; FC: functional connectivity; HC: healthy controls; IOG: inferior occipital gyrus; MOG: middle occipital gyrus; MTG: middle temporal gyrus; PSAC: post‐stroke with abnormal cognition; PSNC: post‐stroke with normal cognition; REC: gyrus rectus; ROIs: regions of interests.

Brain areas with significant ReHo changes were designated as ROIs, including MTG, ITG, MFG, REC, SFG, PoCG, SMA, and CBL IX (Figure S3). Only the left REC, ROI showed significant FC alterations between the two‐stroke groups (Table S3). Compared to HC, PSAC showed increased FC between the left REC and left inferior occipital gyrus (IOG) (Figure 2A; Table 3). PSNC had increased FC between left REC and left middle occipital gyrus (MOG), right REC, and left calcarine fissure (CAL) (Figure 2B; Table 3). When PSAC compared to PSNC, FC was significantly decreased between left REC and right REC and left MTG (Figure 2C; Table 3).

**TABLE 3:** The differences of FC based on ROIs of left REC by post hoc analyses among the three groups.

Brain region (AAL)	Peak MNI coordinate			Peak T‐value	Cluster size
	*x*	*y*	z		
**PSAC > HC**					
Left inferior occipital gyrus	−45	−72	−9	4.7198	60
**PSNC > HC**					
Left middle occipital gyrus	−42	−63	3	6.6043	1453
Right gyrus rectus	6	24	−15	5.0731	139
Left calcarine fissure and surrounding cortex	−6	−69	12	4.4645	105
**PSAC < PSNC**					
Right gyrus rectus	12	39	−21	−3.743	47
Left middle temporal gyrus	−48	−60	15	−4.0370	46

*Note*: The resultant T‐maps were conducted with Gaussian random field theory (GRF) correction for multiple comparisons with voxel *p* < 0.01, cluster *p* < 0.05, and cluster size > 30 voxels.

Abbreviations: AAL: automated anatomical labeling; FC: functional connectivity; HC: healthy controls; MNI: Montreal Neurological Institute; PSAC: post‐stroke with abnormal cognition; PSNC: post‐stroke with normal cognition; REC: gyrus rectus; ROIs: regions of interests.

### 3.4 Association Between Changes in ReHo or FC and Global Cognitive Function Scores

**FIGURE 3:**
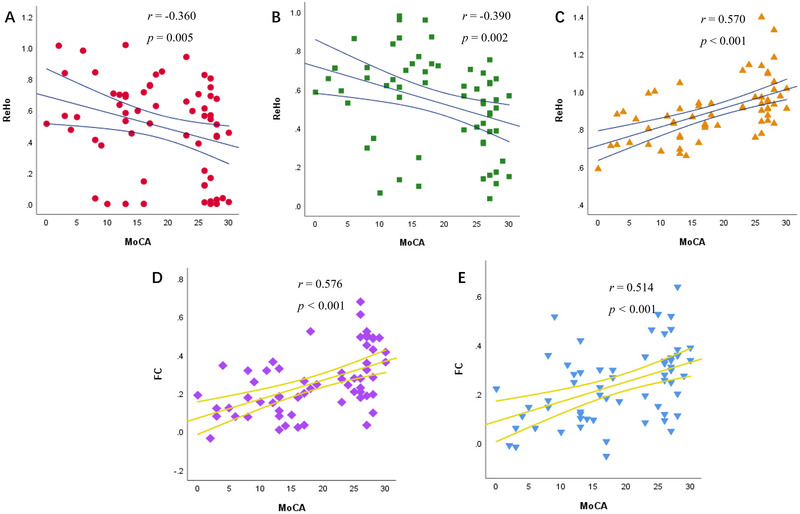
The correlations between (A) the mean ReHo value in the left CBL IX, (B) the mean ReHo value in the right CBL VIII, (C) the mean ReHo value in the left REC, (D) the FC between left REC and right REC, and (E) the FC between left REC and left MTG and MoCA scores in all stroke patients. CBL: cerebellum lobules; FC: functional connectivity; MoCA: Montreal Cognitive Assessment; MTG: middle temporal gyrus; REC: gyrus rectus; ReHo: regional homogeneity.

Partial correlation analysis showed that MoCA scores in stroke groups were negatively correlated with ReHo values in left CBL IX (*r* = −0.360, *p* = 0.005, Figure 3A) and right CBL VIII (*r* = −0.390, *p* = 0.002, Figure 3B). Altered ReHo in left REC is positively correlated to MoCA (*r* = 0.570, *p* < 0.001, Figure 3C). FC between left and right REC (*r* = 0.576, *p* < 0.001, Figure 3D) and between left REC and left MTG (*r* = 0.514, *p* < 0.001, Figure 3E) also showed positive correlations with MoCA scores.

## 4 Discussion

In this study, patients with and without cognitive impairment who suffered non‐acute strokes showed distinct aberrations in brain function utilizing ReHo and data‐driven seed‐based FC. These findings may enhance our understanding of the neuro‐pathophysiological causes of cognitive impairment in post‐stroke patients.

The first interesting finding was that PSAC group had ReHo value decreased in left REC than PSNC. The REC is a component of the orbitofrontal cortex (OFC) in the medial prefrontal cortex (mPFC) subregion, situated medial to the sulcus bromide at the base of the frontal lobe (Szczepanski and Knight 2014). Studies on specific species have demonstrated that different forms and degrees of cognitive impairment are displayed when the mPFC is damaged (Szczepanski and Knight 2014; Wallis 2011). Among them, the OFC is involved in inhibitory control, and decision‐making, emotion and social behavior control (Clark et al. 2008; Dias et al. 1996; Hornak et al. 2003; Piras et al. 2023). However, fewer studies have been conducted only for REC.

Neurosurgeons have focused on cognitive dysfunction in subarachnoid hemorrhage patients, particularly those with anterior communicating artery aneurysms (ACoA), who frequently exhibit memory loss (Chalouhi et al. 2013; Al‐Khindi et al. 2010) and personality changes post‐surgery. The rupture and hemorrhage may directly damage the REC and orbital gyrus at the frontal lobe base (Chalouhi et al. 2013). Specialists have noted temporary and long‐term negative effects on language and memory recall post‐REC resection for ruptured ACoA (Pereira‐Filho et al. 2014; Joo et al. 2016), indicating susceptibility to cognitive issues. REC excision, previously thought not to affect limb movement or sensory function, is linked to cognitive problems. Studies show decreased REC gray volume but increased ReHo in VCI, highlighting REC's cognitive role (Cai et al. 2023). Gao et al. (2021) found higher ReHo in left REC in non‐dominant hemisphere acute stroke patients, linked to motor modulation but not cognition. This research advances the understanding of REC activity in stroke and cognitive functions, necessitating further studies to explore REC function comprehensively.

The study identifies cerebellar abnormalities linked to cognitive functions such as sensorimotor control, language, spatial, emotional, and executive functions (Stoodley and Schmahmann 2009; Stoodley et al. 2012). Activation in the posterior lobes (lobules VI–IX) is associated with visuospatial working memory and language activities (Stoodley and Schmahmann 2009). Previous studies reported reduced ReHo or low‐frequency fluctuation (fALFF) in cerebellar infarction and VaMCI patients compared to HC, while VCI patients showed higher ReHo in the left posterior cerebellum negatively correlated with MoCA scores (Fan et al. 2019; Zuo et al. 2018). Our findings align with those in VCI, highlighting the cerebellum's role in post‐stroke cognitive performance (Diciotti et al. 2017; Zhuang et al. 2021). However, the causes of cerebellar dysfunction and cognitive issues in subcortical strokes remain unclear. Notably, increased FC in the cerebellar anterior lobe with regions like the prefrontal cortex and in the posterior lobe with regions such as the inferior parietal lobule correlates with executive and memory functions in chronic stroke patients (Hong et al. 2022). Further exploration of neuroimaging mechanisms is needed.

Moreover, ReHo values in left REC were positively correlated with MoCA and negatively in the left and right CBL in all stroke patients. In other words, the lower the ReHo of the left REC and the higher the ReHo of the CB, the more severe the cognitive function deficit. Consequently, we propose that the two groups show different patterns of spontaneous brain activity, and the left REC and bilateral cerebellar lobules are not common sites of injury but are intimately associated with GCF.

A control group was included to confirm previous findings (Cai et al. 2023; Szczepanski and Knight 2014; Dias et al. 1996; Joo et al. 2016; Stoodley and Schmahmann 2009) and compare cognitively normal stroke survivors with healthy individuals. Both stroke groups displayed decreased ReHo in the left cerebellum IX and increased ReHo in the frontal and temporal cortices compared to controls. Notably, PSNC cases exhibited abnormal brain activity in PoCG and SMA, with more spontaneous activity fluctuations than cognitively impaired patients. This may result from compensatory dynamics in the brain regions, the inclusion of patients mostly in the subacute stage, and motor or swallowing dysfunctions in the cognitively normal group. Further, longitudinal studies are needed to assess changes in the chronic phase.

For FC analysis, we used data‐driven seed‐based whole‐brain connectivity instead of conventional independent component analysis. The FC of REC is still not well described, though it is part of the mPFC—a key DMN node (Zhuang et al. 2021). Studies in “first‐ever” post‐stroke patients show conflicting cognitive performance results linked to mPFC and brain areas like the hippocampus, MTG, and PCC (Dacosta‐Aguayo et al. 2015; Tuladhar et al. 2013; Zhu et al. 2017). Even subcortical or brainstem strokes exhibited decreased FC of mPFC in the anterior DMN (H. Chen et al. 2019). The mPFC, connected to circuits like striato‐pallido‐thalamo‐cortical and frontostriatal, may have altered connectivity due to subcortical lesions (Benjamin et al. 2014; Orth et al. 2022). This suggests that patients with abnormal FC of REC in subcortical strokes are prone to cognitive impairment, consistent with the understanding of DMN, PCC, and cognition (Dacosta‐Aguayo et al. 2014; Peng et al. 2016). In addition, the mPFC, including the REC, may affect visuospatial attention, as shown by reduced FC with dorsal attention network nodes and symptom regression (Cao et al. 2022), aligning with our findings.

MTG plays a crucial role in language and semantic memory processing (Papeo et al. 2019; Kable et al. 2005), and left hemisphere injury often leads to apraxia (Timpert et al. 2015). MTG, potentially involving the left posterior MTG is also an important node in the DMN (De Renzi and Lucchelli 1988), or MTG‐SMA connection disruption (Ramayya et al. 2010). Multimodal imaging reveals reduced grey matter in MTG in VaMCI patients (Stebbins et al. 2008) and altered ReHo or ALFF in VCI (Cai et al. 2023; Li et al. 2015). Stroke patients show higher ReHo in MTG, suggesting compensatory activity (Liu et al. 2017; Sun et al. 2011; Yi et al. 2012; Li et al. 2021). FC between left MTG and some frontal brain regions were also significantly decreased in VaMCI (Li et al. 2021). We thus hypothesized that PSAC had lower FC levels in DMN, which was also consistent with previous studies (Dacosta‐Aguayo et al. 2014; Dacosta‐Aguayo et al. 2015; Tuladhar et al. 2013; H. Chen et al. 2019).

Combining ReHo and FC results, spontaneous brain activity and FC were different within two stroke groups, suggesting that abnormalities in these brain areas and connection with other brain regions after stroke strongly assume cognitive decline. Meanwhile, the ReHo and FC value is significantly correlated with clinical performance. Previous studies have shown that post‐stroke patients with cognitive deterioration had less consistent localized spontaneous brain activity in bilateral ACC, left PCC, precuneus, and left occipital lobe regions than cognition normal stroke survivors (Peng et al. 2016). In terms of FC, post‐stroke patients with cognitive loss had higher executive control network and basal ganglia network FC but lower DMN and frontotemporal network connection (Dacosta‐Aguayo et al. 2014).

The link between lesion sites and cognitive impairment after stroke is debated, as these sites often involve cortical regions and the thalamus but do not fully predict cognitive outcomes (Weaver et al. 2021). Machine‐learning models identify the left angular gyrus, left basal ganglia, and surrounding white matter as predictors of global cognitive impairment 3–6 months post‐ischemic stroke (Zhao et al. 2018). Some suggest stroke lesions indirectly affect whole‐brain function (Veldsman et al. 2020). In this study, 67.7% of patients had basal ganglia lesions, with balanced distribution across stroke groups, suggesting that reduced spontaneous activity and connectivity in the left REC may be key factors in subcortical stroke‐related cognitive loss. Spontaneously, the cerebellum may compensate for some functional decline. These findings propose the left REC as a potential target for neuroregulatory intervention. Moreover, pre‐stroke cognitive performance and cerebral small vessel disease can predispose individuals to cognitive decline, post‐stroke cognitive impairment (PSCI), or post‐stroke dementia (PSD) (Diciotti et al. 2017; McMurtray et al. 2007; Pendlebury and Rothwell 2009; Markus and de Leeuw 2023). Future studies should include diffusion tensor imaging to explore white matter conduction in PSCI and conduct longitudinal research to identify PSCI and PSD risk factors through neuroimaging.

### 4.1 Limitations

Our study has several limitations. First, differences in educational level and stroke type were present between the stroke groups. Previous research indicates that lower education levels are associated with higher risks of PSCI and PSD after stroke, although it's unclear if education is an independent risk factor for dementia (Yang et al. 2015; Filler et al. 2024). Regarding stroke type, no significant differences in brain activity and FC have been reported between hemorrhagic and ischemic strokes (Wang, Rao, Yue, et al. 2021). Future research should address these findings. Second, the cross‐sectional design limits the detection of dynamic changes in ReHo and FC post‐stroke, highlighting the need for long‐term studies. Third, the small sample sizes in each group, similar to other studies with 20–35 cases (Peng et al. 2016; Wang, Rao, Chen, et al. 2021; Liu et al. 2021), necessitate replication in larger, longitudinal studies for validation. Lastly, our correlation analysis focused only on overall cognitive function, indicating the need for more specific assessments across different cognitive domains.

## 5 Conclusion

In summary, the PSAC group exhibited distinct patterns of brain activity and reduced FC compared to the PSNC group. These neural alterations were strongly associated with cognitive function and may underlie cognitive impairment following subcortical non‐acute stroke. Our findings highlight the left REC as the potential target for neuromodulation and deepen our understanding of the neuropathophysiological mechanisms involved in post‐stroke cognitive impairment.

## Author Contributions

**Yao Wang**: Software, data curation, writing – original draft, funding acquisition, formal analysis, investigation, visualization, resources, conceptualization, writing – review and editing, validation. **Wan Liu**: Methodology, software, investigation, validation, visualization, writing – review and editing. **Wenjie Yang**: Data curation, investigation. **Xue Chai**: Methodology, software, data curation. **Hao Yu**: Methodology, project administration, software. **Hongxia Ma**: Methodology, project administration, data curation, writing – review and editing. **Li Liu**: Conceptualization, investigation, project administration, supervision, resources. **Jiang Rao**: Conceptualization, investigation, funding acquisition, writing – review and editing, methodology, project administration, data curation, resources. **Guangxu Xu**: Conceptualization, project administration, resources, supervision, data curation, writing – review and editing. **Zhibin Hu**: Conceptualization, methodology, supervision, project administration, writing – review and editing, data curation.

## Conflicts of Interest

The authors declare no conflicts of interest.

### Peer Review

The peer review history for this article is available at https://publons.com/publon/10.1002/brb3.70356.

## Ethics Statement

All experiments in this study were conducted in accordance with the Declaration of Helsinki and that all procedures were carried out with the adequate understanding and written consent of the subjects. The Nanjing Medical University Affiliated Brain Hospital ethics committee approved this study (No. 2022‐KY086‐01).

## Consent

All participants gave written informed consent.

## Supporting information



Supporting Information

## Data Availability

The datasets generated during and/or analyzed during the current study are available from the corresponding author upon reasonable request.
