# Supplementary material for: Differential Abnormality in Regional Brain Spontaneous Activity and Functional Connectivity in Patients of Non‐Acute Subcortical Stroke With Versus Without Global Cognitive Functional Impairment
Source: Brain Behav. 2025 Feb 25;15(2):e70356. doi: 10.1002/brb3.70356 (PMC11860280; doi:10.1002/brb3.70356)
Supplement: Supplementary file 1 — Supporting Information [file BRB3-15-e70356-s001.zip › brb370356-sup-0001-SuppMat/Supplemental files/Supplemental Table1.docx]

Supplemental Table 1. TOAST subtypes for ischemic stroke in groups of PSAC and PSNC.

|  | **PSAC (n=32)** | **PSNC (n=30)** | ***χ*^2^** | ***p* value** |
| --- | --- | --- | --- | --- |
| TOAST subtypes  Large-artery atherosclerosis  Small-artery occlusion | 18  0 | 24  2 | 1.451 | 0.228 |

PSAC: Post-Stroke with Abnormal Cognition; PSNC: Post-Stroke with Normal Cognition; TOAST: Trial of Org 10172 in Acute Stroke Treatment.
