# Supplementary material for: Differential Abnormality in Regional Brain Spontaneous Activity and Functional Connectivity in Patients of Non‐Acute Subcortical Stroke With Versus Without Global Cognitive Functional Impairment
Source: Brain Behav. 2025 Feb 25;15(2):e70356. doi: 10.1002/brb3.70356 (PMC11860280; doi:10.1002/brb3.70356)
Supplement: Supplementary file 1 — Supporting Information [file BRB3-15-e70356-s001.zip › brb370356-sup-0001-SuppMat/Supplemental files/Supplemental Table2.docx]

Supplemental Table2. The differences in the brain regions of ReHo among the three groups (ANCOVA).

| Region (AAL) | Peak MNI coordinate | | | Peak F value | Cluster size |
| --- | --- | --- | --- | --- | --- |
|  | x | y | z |  |  |
| **ANCOVA** |  |  |  |  |  |
| Left Cerebelum_9 | -12 | -45 | -54 | 14.8713 | 873 |
| Left Inferior temporal gyrus/ Left Middle temporal gyrus | -63 | -30 | -21 | 18.2479 | 341 |
| Right Inferior temporal gyrus /Right Middle temporal gyrus | 66 | -33 | -18 | 16.1553 | 316 |
| Left Middle frontal gyrus/ Left Gyrus rectus | -24 | 48 | -15 | 18.7429 | 577 |
| Right Superior frontal gyru | 18 | 24 | -18 | 18.9194 | 307 |
| Right Anterior cingulate and paracingulate gyri/ R Supplementary motor area | 9 | 42 | 18 | 12.7581 | 191 |
| Right Postcentral gyrus | 66 | -12 | 33 | 12.2144 | 271 |
| Left Middle frontal gyrus | -33 | 21 | 42 | 19.3354 | 443 |

AAL, automated anatomical labeling; ANCOVA: Analysis of covariance; MNI, Montreal Neurological Institute; ReHo: Regional homogeneity.

The resultant T-maps were conducted with Gaussian Random Field Theory (GRF) correction for multiple comparisons with voxel *p* < 0.01, cluster *p* < 0.05, and cluster size >50 voxels.
