# Supplementary material for: Differential Abnormality in Regional Brain Spontaneous Activity and Functional Connectivity in Patients of Non‐Acute Subcortical Stroke With Versus Without Global Cognitive Functional Impairment
Source: Brain Behav. 2025 Feb 25;15(2):e70356. doi: 10.1002/brb3.70356 (PMC11860280; doi:10.1002/brb3.70356)
Supplement: Supplementary file 1 — Supporting Information [file BRB3-15-e70356-s001.zip › brb370356-sup-0001-SuppMat/Supplemental files/Supplemental Table3.docx]

Supplemental Table 3. The differences of FC based on ROI of left REC among the three groups.

| **Brain Region (AAL)** | Peak MNI coordinate | | | Peak F-value | Cluster size |
| --- | --- | --- | --- | --- | --- |
|  | x | y | z |  |  |
| **ANCOVA** |  |  |  |  |  |
| Left Middle occipital gyrus/ Left Middle temporal gyrus/Left Inferior occipital gyrus | -39 | -66 | 3 | 19.8362 | 1475 |
| Right Superior frontal gyrus**/**Right Gyrus rectus | 6 | 24 | -12 | 14.3799 | 157 |
| Left Calcarine fissure and surrounding cortex | -6 | -69 | 12 | 8.7203 | 107 |

AAL: Automated Anatomical Labeling; ANCOVA: Analysis of covariance; FC: Functional Connectivity; MNI, Montreal Neurological Institute; REC: Gyrus Rectus ROI: Regions of Interests.

The resultant T-maps were conducted with Gaussian Random Field Theory (GRF) correction for multiple comparisons with voxel *p* < 0.01, cluster *p* < 0.05, and cluster size > 30 voxels.
