# Supplementary material for: Differential Abnormality in Regional Brain Spontaneous Activity and Functional Connectivity in Patients of Non‐Acute Subcortical Stroke With Versus Without Global Cognitive Functional Impairment
Source: Brain Behav. 2025 Feb 25;15(2):e70356. doi: 10.1002/brb3.70356 (PMC11860280; doi:10.1002/brb3.70356)
Supplement: Supplementary file 1 — Supporting Information [file BRB3-15-e70356-s001.zip › brb370356-sup-0001-SuppMat/Supplemental files/Supplementary Material-file1.docx]

**Brain MRI Data Acquisition**

MRI scanning was acquired with a Siemens 3.0-T signal scanner (Siemens, Verio, Germany) in Affiliated Brain Hospital of Nanjing Medical University. All subjects lay supine with their head fixed by foam pads with a standard birdcage head coil to minimize head movement. Participants were instructed to remain as still as possible, open their eyes remain awake, and not think of anything. High-resolution T1-weighted images were acquired by 3D magnetization-prepared rapid gradient-echo (MPRAGE) sequence (repetition time [TR] =1,900ms; echo time [TE] =2.48ms; flip angle [FA] =9 degrees; matrix =256×256; field of view [FOV] =256×256 mm^2^ ; slice thickness/gap =1/0.5 mm; 176 slices covered the whole brain) for image registration and functional localization. The imaging took approximately 260 seconds. Functional images were subsequently collected in the same slice orientation with a gradient-recalled echo-planar imaging pulse sequence (TR= 2,000ms; TE =30ms; FA =90degrees; matrix= 64]×64, FOV= 220×220mm^2^ ; thickness/gap =4.0/0mm;voxel size = 3.4×3.4×4 mm^3^ ; slice numbers =36). A total of 240 volumes were obtained in this acquisition sequence and each functional resting-state session lasted 480 seconds.
