# Supplementary figures and images for: Differential Abnormality in Regional Brain Spontaneous Activity and Functional Connectivity in Patients of Non‐Acute Subcortical Stroke With Versus Without Global Cognitive Functional Impairment
Source: Brain Behav. 2025 Feb 25;15(2):e70356. doi: 10.1002/brb3.70356 (PMC11860280; doi:10.1002/brb3.70356)

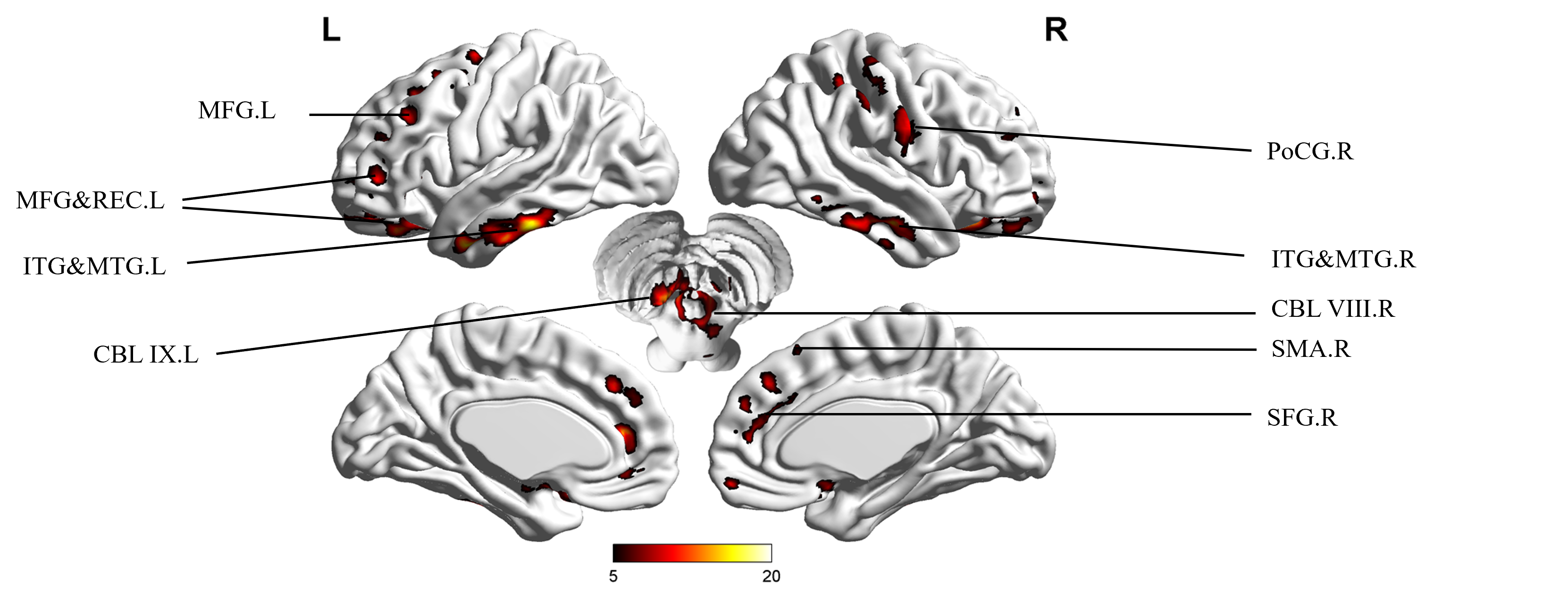

Supplement: Supplementary file 1 — Supporting Information [file BRB3-15-e70356-s001.zip › brb370356-sup-0001-SuppMat/Supplemental files/Suppemental Figure3.tif]

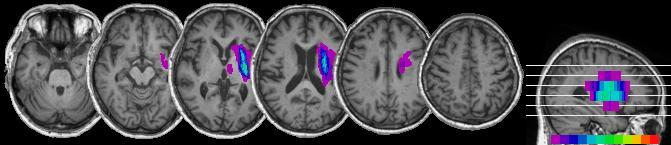

Supplement: Supplementary file 1 — Supporting Information [file BRB3-15-e70356-s001.zip › brb370356-sup-0001-SuppMat/Supplemental files/Supplemental figure 1/Supplemental Figure1C_PSAC with left lesions.tif]

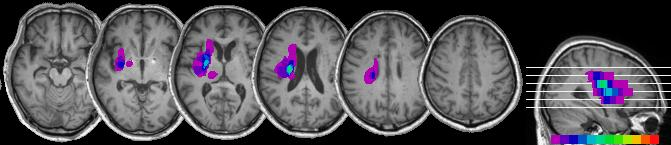

Supplement: Supplementary file 1 — Supporting Information [file BRB3-15-e70356-s001.zip › brb370356-sup-0001-SuppMat/Supplemental files/Supplemental figure 1/Supplemental Figure1D_PSAC with right lesions.tif]

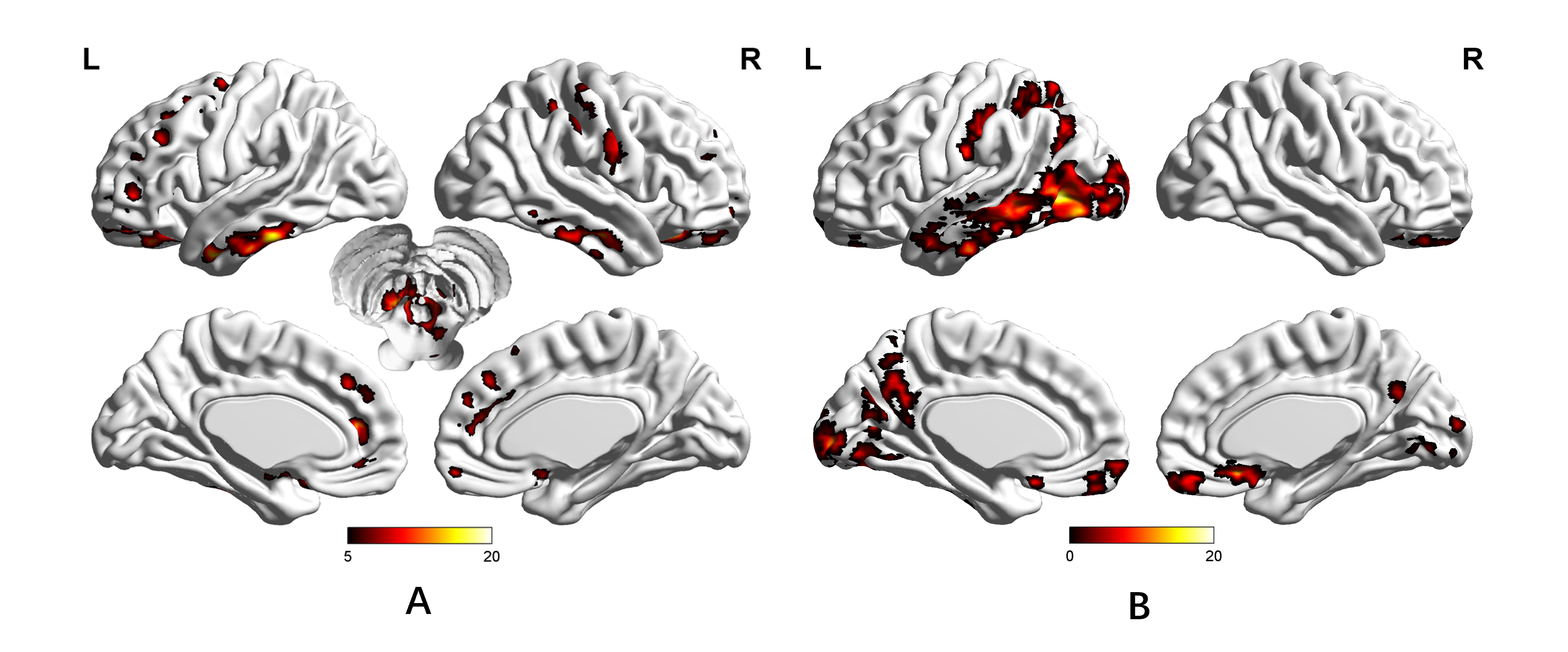

Supplement: Supplementary file 1 — Supporting Information [file BRB3-15-e70356-s001.zip › brb370356-sup-0001-SuppMat/Supplemental files/Supplemental Figure2.tif]
